# Letter to the Editor regarding “Development of an antibody-free ID-LC MS method for the quantification of procalcitonin in human serum at sub-microgram per liter level using a peptide-based calibration”

**DOI:** 10.1007/s00216-021-03459-5

**Published:** 2021-07-01

**Authors:** Sebastian-Alexander Tölke, Thomas Masetto, Matthias Grimmler, Laura Bindila, Klaus Schneider

**Affiliations:** 1 Hochschule Fresenius University of Applied Sciences, Limburger Straße 2, 65510 Idstein, Germany; 2 Institut für Molekulare Medizin I, Heinrich-Heine-Universität, Universitätsstr. 1, 40225 Düsseldorf, Germany; 3 DiaSys Diagnostic Systems GmbH, Alte Str. 9, 65558 Holzheim, Germany; 4 Clinical Lipidomics Unit, Institute of Physiological Chemistry, University Medical Center of the JGU Mainz, 55128 Mainz, Germany

Dear editor,

Procalcitonin (PCT) is an important clinical marker for assessing bacterial sepsis [1]. Several routine assays from different manufacturers and of different format are available for its diagnosis and monitoring. The necessity and feasibility for a standardization of PCT quantification have recently been described by Huynh et al. [2]. Efforts to generate a reference measurement system and certified reference materials are on the way as a coordinated undertaking by the working group for PCT standardization of the International Federation of Clinical Chemistry (IFCC) with the participation of the authors of this letter as well as of Huynh et al. [2].

In their article in this journal, Huynh et al. describe a method for PCT quantification by isotope-dilution liquid chromatography tandem mass spectrometry (ID-LC-MS/MS) [3]. The enrichment of PCT out of the complex serum matrix was achieved by two steps of solid-phase extraction (SPE), initially for enrichment of intact PCT from serum and subsequently for capture of PCT peptides generated by trypsin digestion. Stable-isotope-labeled synthetic peptides were used as internal standards, and a limit of quantitation (LOQ) of 0.25 μg/L based on the detection of a single tryptic peptide was demonstrated. While we consider the LOQ encouraging, it does fall short of the diagnostically relevant range for PCT which extends down to 0.05 μg/L. Importantly, the method by Huynh et al. faces several shortcomings and limitations, among which the main points are:

(i) The need for a second SPE step that effectively restricts detection to only one subset of tryptic peptides that are amenable to capture and elution under the chosen SPE conditions. Only two peptides were reported to fulfill these criteria, of which one was used as qualifier and the other for quantitation. Any PCT modification or PCT processing not covered by peptides eluting under these SPE conditions cannot be detected.
(ii) The use of isotope-labeled peptides for standardization may lead to a systematic error due to potentially incomplete PCT digestion. Furthermore, sub-optimal capture and elution of intact PCT in the first SPE step cannot be compensated by addition of peptide internal standard. The reliability of a correction factor aiming to address varying SPE recovery or digestion efficiencies cannot be guaranteed for a complex sample preparation process of this kind.

To achieve the aim of generating a robust reference measurement system, it is imperative to address these shortcomings. Further improvements in standardization will be required while improved detection of PCT can be achieved using different principles for PCT enrichment.

**Fig. 1:**
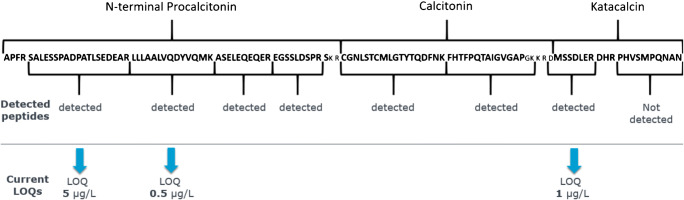
Coverage of PCT protein sequence with tryptic peptides detected by immunoaffinity-LC-MS/MS. LOQs of peptides subsequently evaluated for use in PCT quantifications are indicated

We have developed an immunoaffinity-ID-LC-MS/MS method for PCT based on immunoaffinity enrichment of intact PCT by a polyclonal PCT antibody (pAb) immobilized on polystyrene carrier material (“latex,” Lx). In our method, stable-isotope-labeled recombinant PCT is added as internal standard prior to the immuno-enrichment step and non-labeled recombinant PCT of high purity is used for calibration. After several washing steps of the Lx material and trypsin digestion of the latex-bound antibody-PCT complex, a larger number of PCT peptides are detected (Fig. 1).

In comparison to the method of Huynh et al, the use of an internal PCT protein standard compensates for potential variation in the enrichment of endogenous PCT as well as potential losses in subsequent washing and handling steps. A potential concern that an antibody may have higher enrichment efficiencies for endogenous or for recombinant PCT is much reduced as a polyclonal antibody will have a higher probability of capturing PCT in potentially different conformational states or protein variants (e.g., PCT isoforms, PCT partially complexed by interacting proteins). We also decided to choose a pAb approach, in contrast to most of the existing monoclonal Ab-based assays, to trace back to the original Kryptor method directly, using polyclonal capture Abs [2]. Furthermore, all tryptic peptides with suitable properties in terms of size and retention by the reversed-phase column are available for detection by LC-MS/MS. This allows for a much wider coverage of the PCT protein sequence by the generated peptides. In addition, it also ensures the availability of all the peptides among which the best ones can be chosen for the quantitative assessment, which—beyond the direct aims for a quantitative PCT method—may facilitate further uses of the method to investigate potential PCT polymorphisms, PCT processing, and/or PCT post-translational modifications.

Out of eight tryptic peptides containing more than four amino acids, seven were detected at levels that were at least in the concentration range of PCT at or below 25 μg/L. Two peptides covering the N-terminal region and one peptide representing the C-terminal Katacalcin sequence of PCT were chosen for quantitative method development. After method optimization, the achieved limits of quantification for PCT were 0.5 μg/L from peptide LLLAALVQDYVQMK, 1 μg/L from peptide DMSSDLLR, and 5 μg/L for peptide SALESSPADPATLSEDEAR. Huynh et al. achieved a slightly better level at 0.25 μg/L for the SALESSPADPATLSEDEAR peptide. This was, however, achieved at lower flow rates of 80 μL/min compared to 300 μL/min in our study. Compared to current routine assays and the diagnostically relevant PCT range starting at 0.05 μg/L [2, 4], both ID-LC-MS/MS methods fall short of the required sensitivity for covering the full range of diagnostically relevant PCT concentrations. While the two methods do utilize different initial steps in enrichment (SPE vs immuno-enrichment), subsequent distinct ID-LC-MS/MS techniques were independently employed by the two groups to detect and quantify PCT. Independent use of ID-LC-MS/MS analysis also proofs methodical cross-validity and robustness for extended comparative studies with routine clinical assays. Finally, we see clear potential of our immunoaffinity method in future sensitivity improvements to 0.1–0.05 μg/L. Prospective achievement of such a sensitivity limit, achievable by reducing LC flow rates and taking advantage of further improvement in detection sensitivities in state-of-the art mass spectrometers, is necessary to better cover and facilitate the decision point of PCT routine diagnostic.
